# COVID-19 Usurps Host Regulatory Networks

**DOI:** 10.3389/fphar.2020.01278

**Published:** 2020-08-14

**Authors:** Colleen S. Curran, Donna R. Rivera, Jeffrey B. Kopp

**Affiliations:** ^1^Critical Care Medicine Department, Clinical Center, National Institutes of Health, Bethesda, MD, United States; ^2^Surveillance Research Program, Division of Cancer Control and Population Sciences, National Cancer Institute, National Institutes of Health, Rockville, MD, United States; ^3^Kidney Disease Section, National Institute of Diabetes and Digestive and Kidney Diseases, National Institutes of Health, Bethesda, MD, United States

**Keywords:** SARS-CoV-2, renin-angiotensin-aldosterone system, COVID-19, pharmacotherapy, angiotensin II, bradykinin, coagulation, substance P

## Abstract

Severe acute respiratory syndrome coronavirus 2 (SARS-CoV-2) infection causes coronavirus disease 2019 (COVID-19). SARS-CoV-2 binds the angiotensin-converting enzyme 2 (ACE2) on the cell surface and this complex is internalized. ACE2 serves as an endogenous inhibitor of inflammatory signals associated with four major regulator systems: the renin-angiotensin-aldosterone system (RAAS), the complement system, the coagulation cascade, and the kallikrein-kinin system (KKS). Understanding the pathophysiological effects of SARS-CoV-2 on these pathways is needed, particularly given the current lack of proven, effective treatments. The vasoconstrictive, prothrombotic and pro-inflammatory conditions induced by SARS-CoV-2 can be ascribed, at least in part, to the activation of these intersecting physiological networks. Moreover, patients with immune deficiencies, hypertension, diabetes, coronary heart disease, and kidney disease often have altered activation of these pathways, either due to underlying disease or to medications, and may be more susceptible to SARS-CoV-2 infection. Certain characteristic COVID-associated skin, sensory, and central nervous system manifestations may also be linked to viral activation of the RAAS, complement, coagulation, and KKS pathways. Pharmacological interventions that target molecules along these pathways may be useful in mitigating symptoms and preventing organ or tissue damage. While effective anti-viral therapies are critically needed, further study of these pathways may identify effective adjunctive treatments and patients most likely to benefit.

## Introduction

The first cases of coronavirus disease 2019 (COVID-19), the infectious disease caused by the severe acute respiratory syndrome coronavirus 2 (SARS-CoV-2), appeared in China in late 2019 (Chen Z. L. et al., 2020). Although SARS-CoV-2 likely originated from an animal host in China (Zhang T. et al., 2020), regular and seasonal travel patterns of individuals near the source allowed the virus to spread globally, prompting a pandemic declaration by the World Health Organization in March 2020 (Chen Z. L. et al., 2020; Sohrabi et al., 2020).

**Figure 1:**
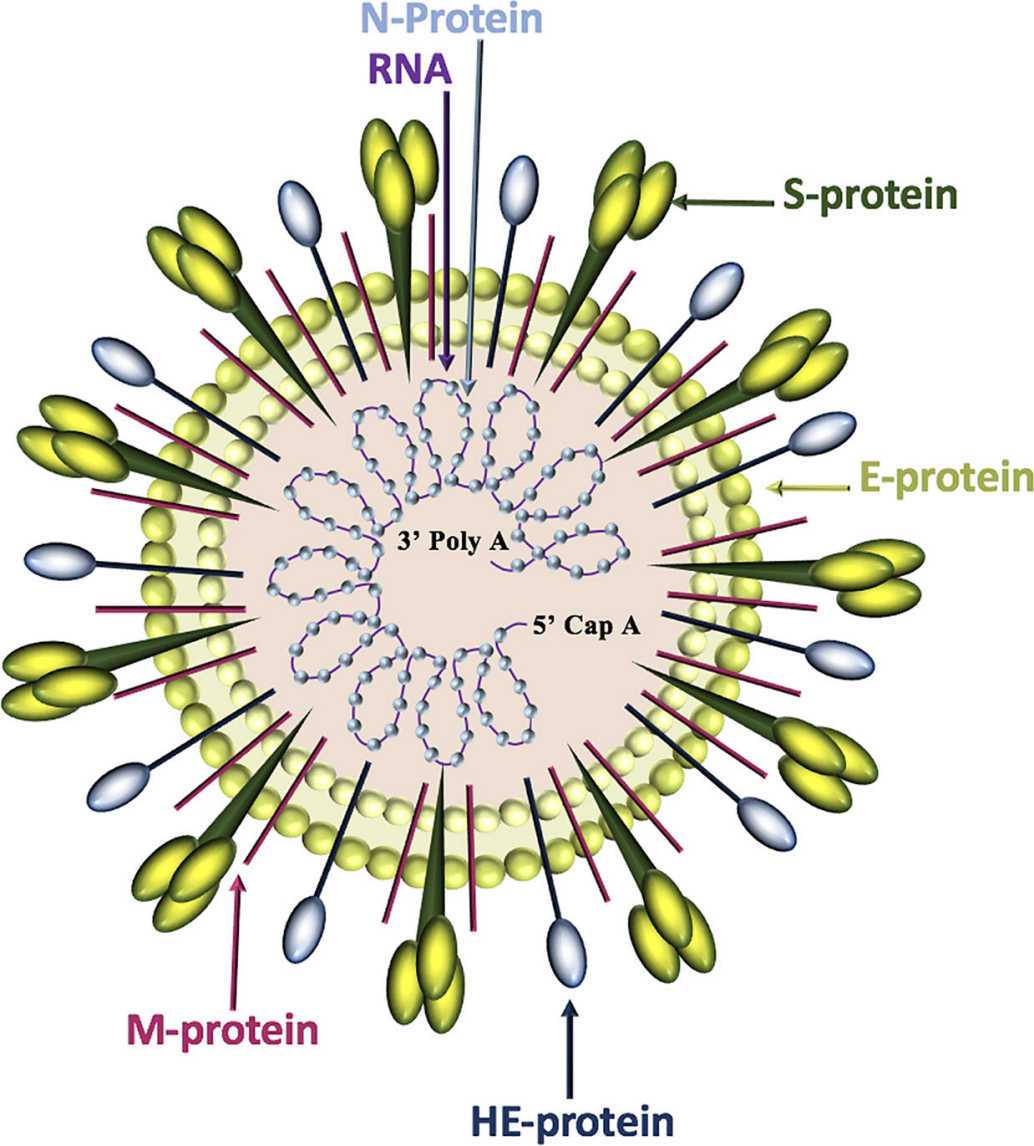
Coronavirus structure. Coronaviruses contain a trimeric spike (S)-protein that mediates attachment to the host receptor, abundant membrane (M)-proteins, envelope (E)-proteins that facilitate assembly and release of the virus, nucleocapsid (N)-proteins that bind positive-strand RNA, and hemagglutinin-esterase (HE)-proteins that bind sialic acids on surface glycoproteins and manifest acetyl-esterase activity.

Of the four genera of coronaviruses *(Alphacoronavirus, Betacoronavirus, Gammacoronavirus, Deltacoronavirus)*, three affect mammals *(Alphacoronavirus, Betacoronavirus, Deltacoronavirus)*, and within the first two genera, seven known human coronaviruses (HCoV) are identified (*Alphacoronavirus*: HCoV-NL63, HCoV-229E; *Betacoronavirus*: SARS-CoV, SARS-CoV-2, MERS-CoV, HCoV-OC43, HCoV-HKU1) (Dominguez et al., 2014). Coronavirus genomes are continuous, single-stranded, non-segmented, positive-sense RNA molecules that contain a 5’ cap and a 3’ poly-A tail for efficient translation by eukaryotic host ribosomes. The four main coronavirus structural proteins are spike (S)-protein, membrane (M)-protein, envelope (E)-protein, and nucleocapsid-(N)-protein, while a fifth protein called hemagglutinin-esterase (HE)-protein is present in a subset of *Betacoronaviruses* (Fehr and Perlman, 2015) (**Figure 1**).

**Figure 2:**
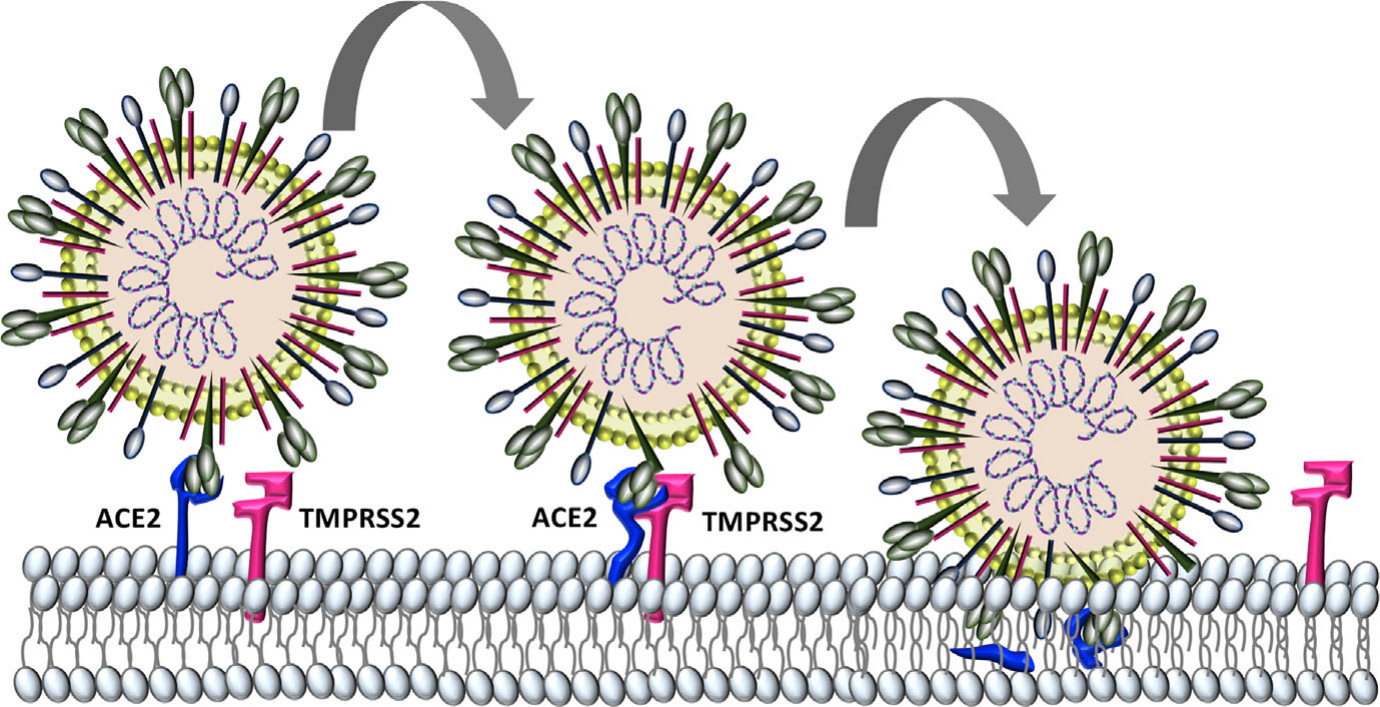
Characterized SARS-CoV-2 entry. SARS-CoV-2 binds to host cell angiotensin converting enzyme-2 (ACE2). The protease TMPRSS2 is recruited and cleaves ACE2 and activates the S protein for membrane fusion and viral entry.

Similar to the human coronaviruses HCoV-NL63 and SARS-CoV, SARS-CoV-2 binds angiotensin-converting enzyme 2 (ACE2) on host cell plasma membranes (Wu et al., 2009; Hoffmann et al., 2020). SARS-CoV-2, and all other human coronaviruses, can also use transmembrane protease, serine 2 (TMPRSS2, epitheliasin) (Milewska et al., 2018; Shirato et al., 2018; Widagdo et al., 2019; Hoffmann et al., 2020), which cleaves ACE2 and activates the S-protein for membrane fusion and viral internalization (Glowacka et al., 2011; Heurich et al., 2014) (**Figure 2**). The extensive sequence homology between SARS-CoV-2 and SARS-CoV (Grifoni et al., 2020) allows for informative comparisons in evaluating SARS-Cov-2 pathogenesis and possible therapeutic treatment targets for COVID-19 patients.

COVID-19 patients may be asymptomatic, have mild symptoms, or present with a febrile pneumonia. Three stages have been described: early infection, pulmonary involvement, and systemic hyperinflammation, sometimes with sepsis (Guan et al., 2020; Huang et al., 2020; Siddiqi and Mehra, 2020). The early phases are characterized by variable combinations of fever, fatigue, dry cough, shortness of breath, headache, pharyngitis, rhinorrhea, hemoptysis, nausea, vomiting, abdominal pain, and diarrhea (Guan et al., 2020; Wang D. et al., 2020). Some patients exhibit cutaneous manifestations (Galvan Casas et al., 2020) or a disruption in smell or taste (Sedaghat et al., 2020). Common clinical findings at admission include ground-glass opacity and bilateral patchy shadowing on chest imaging, lymphocytopenia, neutrophilia, and elevated levels of C-reactive protein (CRP) (Chen G. et al., 2020; Chen N. et al., 2020; Guan et al., 2020). Disease progression is most common in patients with hypertension, diabetes, coronary heart disease and immune deficiencies and these populations are also at increased risk of multiple organ failure and death (Kuderer et al., 2020; Zhou et al., 2020). Greater disease severity is associated with higher levels of alanine aminotransferase, interleukin (IL)-6, CRP, tumor necrosis factor (TNF), and fibrin degradation products (D-dimers) as well as markedly lower levels of blood lymphocytes (Chen G. et al., 2020). Multi-system involvement can include cardiovascular, pulmonary, hepatic, and renal effects, as well as COVID-19 manifesting coagulopathies, shock and multiple organ failure.

**Figure 3:**
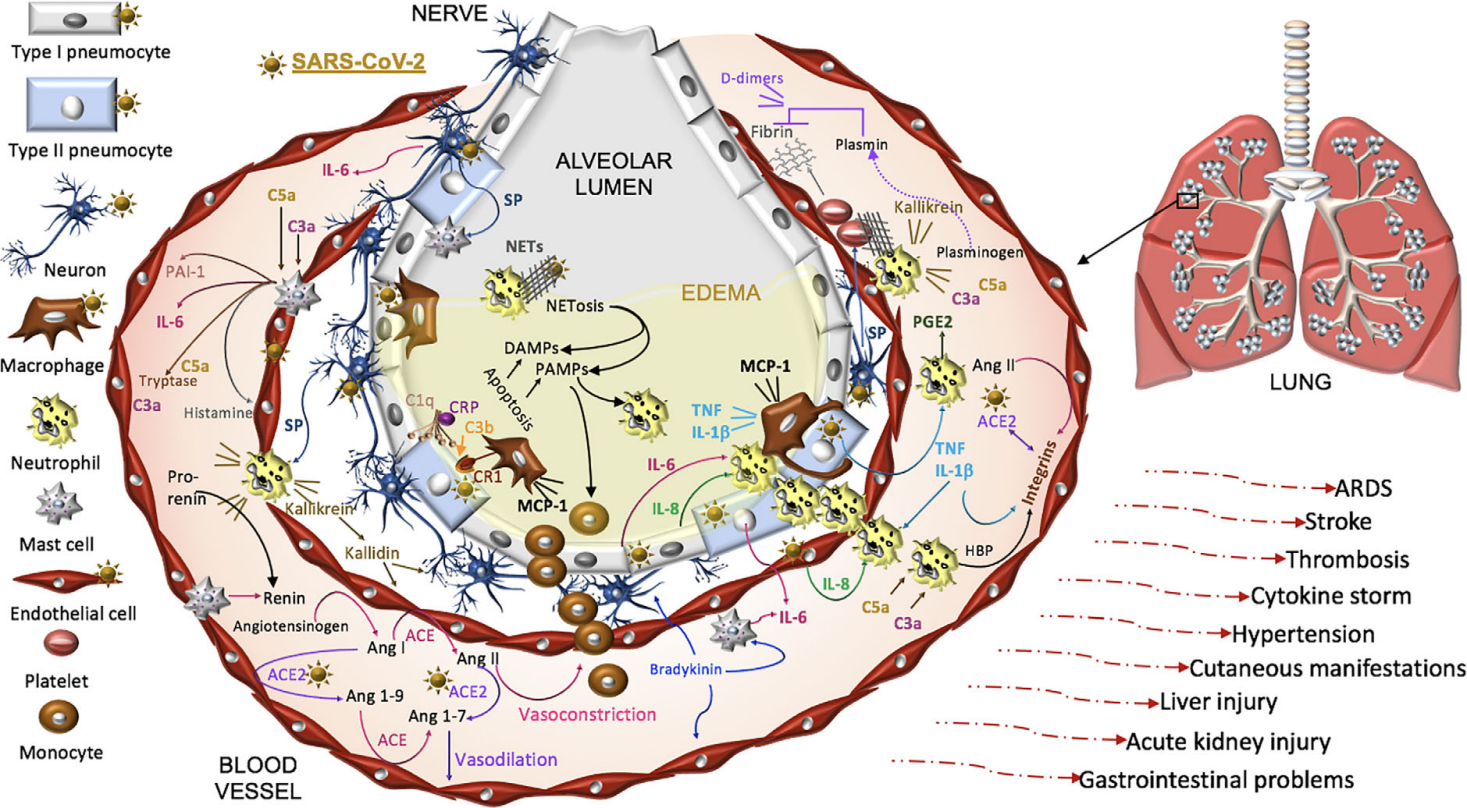
Proposed model of SARS-CoV-2 pathogenesis. SARS-CoV-2 binds ACE2 on alveolar type I and type II cells, macrophages, neurons, and arterial and venous endothelial cells. Complement, damage-and pathogen-associated molecular pattern ligands (DAMPs, PAMPs), and cytokines prime circulating neutrophils that are recruited in response to IL-8 and IL-6 released from infected cells and mast cells. SARS-CoV-2-induces epithelial apoptosis. Alveolar macrophages remove apoptotic cells through complement independent and dependent mechanisms. Apoptotic cells generate antigens that bind C1q and the interaction may be enhanced by C reactive protein (CRP). The activation of C1q induces C3b deposition for macrophage complement receptor 1 (CR1) binding and phagocytosis. Activated neutrophils produce heparin binding protein (HBP), prostaglandins (PGE2), and extracellular traps (NETs) to capture and kill the virus. NET activity induces a form of cell death called NETosis. Both NETosis and apoptosis generate DAMPs and PAMPs that bind and activate toll-like receptors in promoting inflammation. Mast cells and neutrophils are activated by complement factors and the neuropeptide, substance P (SP), which promotes their degranulation. Mast cells also produce histamine that promotes vasodilation, tryptase involved in complement factor production, and renin in the RAAS. Excessive inflammation, associated with MCP-1-recruited monocytes, promotes the accumulation of fluid, leading to alveolar edema. Platelets are activated by SP and exhibit cross-talk with neutrophil NETs in promoting coagulation. The kallikrein-kinin system is activated by damaged tissue and cells, such as neutrophils. Kallikrein functions as a precursor to bradykinin, activates pro-renin, and cleaves complement C3 and C5 as well as plasminogen. The latter generates plasmin involved in the degradation of fibrin and the formation of D-dimers identified in COVID-19 patient serum. SARS-CoV-2-induced degradation of ACE2 promotes RAAS activity, vasoconstriction, and hypertension. Angiotensin II, various cytokines, and HBP induce the expression of endothelial integrins. In the absence of ACE2, which also binds integrins, the functions of integrins may be dysregulated, promoting inflammation, hypertension, and thromboses. The infection can proceed to acute respiratory distress syndrome (ARDS) and culminates in additional tissues and organs in response to systemic infection.

Thus, COVID-19 pathogenesis is complex and while intensive investigations have been made, there is undoubtedly much still to be learned in this is rapidly developing field. Here we focus on SARS-CoV-2 pathophysiology in the renin-angiotensin-aldosterone system (RAAS), the complement pathway, the coagulation pathway, and the kallikrein-kinin system that are each impacted by the primary SARS-CoV-2 receptor, ACE2. Dysfunction in these pathways is linked to three systemic manifestations that occur in many patients with COVID-19. First, patients are at an increased risk for thromboembolism and systemic vasculitis. Second, patients often have low platelet counts, elevated D-dimer levels, prolonged pro-thrombin time and may develop disseminated intravascular coagulation. Third, COVID-19 patients may develop localized pulmonary angioedema, manifesting as fever, dry cough, dyspnea, and in some cases respiratory failure and/or a systemic cytokine storm (**Figure 3**).

## Highlighted Cell Types in SARS-CoV-2 Pathophysiology

### Epithelial Cells

**Figure 4:**
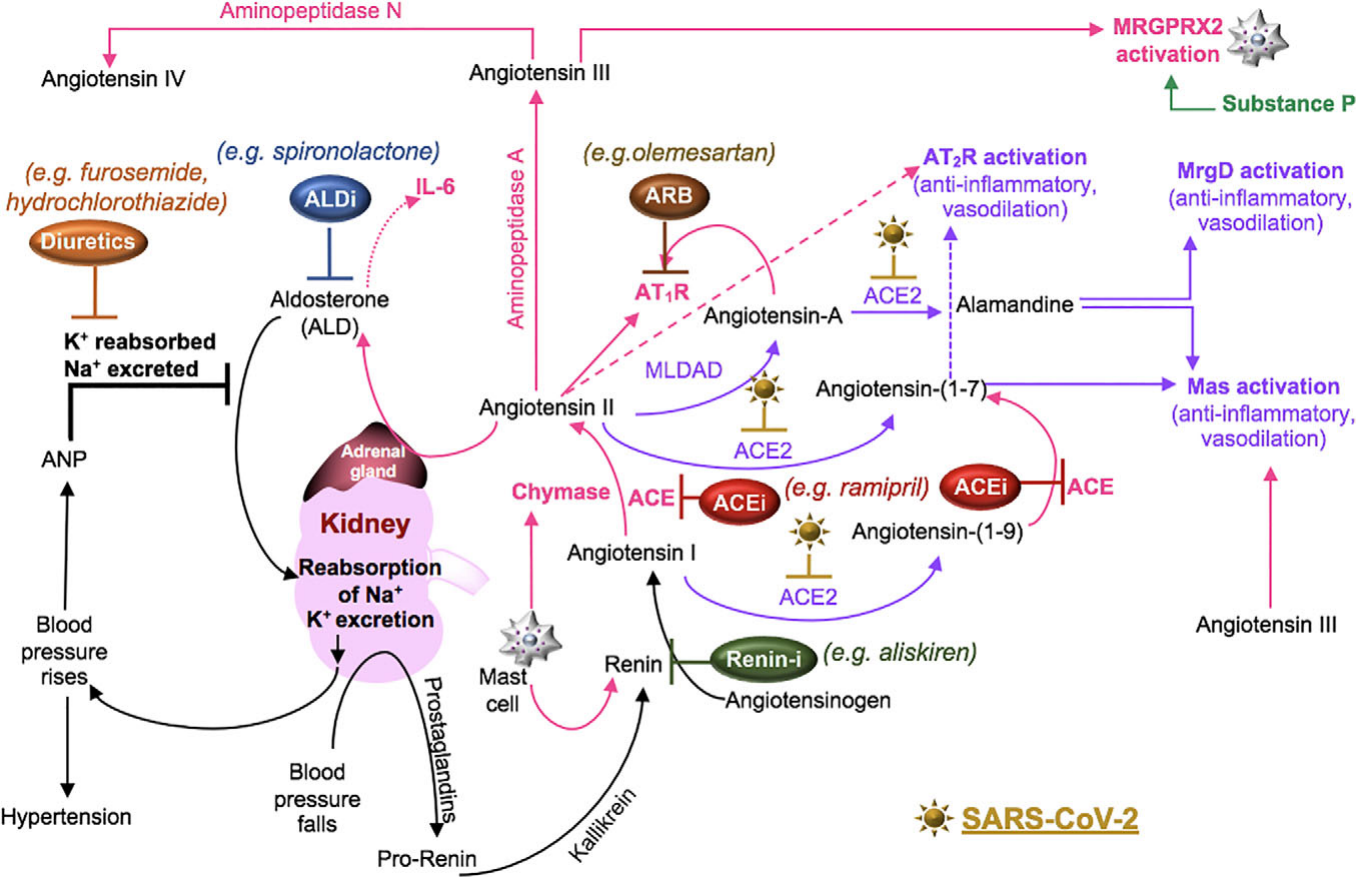
SARS-CoV-2 in the RAAS pathway. Prostaglandins stimulate the release of pro-renin from juxtaglomerular cells. Pro-renin is cleaved to renin by kallikrein. Renin is also produced by activated mast cells. Renin transforms angiotensinogen into angiotensin I. ACE or mast cell chymase converts angiotensin I into angiotensin II, which binds AT_1_R, and stimulates the production of aldosterone and subsequently IL-6. Aldosterone promotes renal distal tubular reabsorption of sodium, increasing blood pressure. In response to intravascular volume expansion, cells in the atrial wall release atrial natriuretic peptide (ANP), which down-regulates angiotensin II activity. Mononuclear leukocyte-derived aspartate decarboxylase (MLDAD) converts the octapeptide angiotensin II to another octapeptide, angiotensin A, which promotes the activation or AT_1_R, or may generate the anti-inflammatory heptapeptide (seven amino acid) ligand, alamandine, *via* ACE2 activity. ACE2 also converts angiotensin II to angiotensin-(1-7), which is a ligand for both the Mas receptor and AT_2_R involved in vasodilation and anti-inflammatory responses. Additional aminopeptidases convert angiotensin II into angiotensin III and IV. Angiotensin III can bind Mas and both angiotensin III and substance P activate MGRPRX2 on mast cells. A lack of ACE2 in RAAS due to SARS-CoV-2-induced degradation may suggest a benefit for intervention along the ACE/angiotensin II/AT_1_R/aldosterone pathway. These may include renin inhibitors (Renin-i), AT_1_R blockers (ARB), ACE inhibitors (ACEi), aldosterone blockers (ALDi), or diuretics.

Cell surface receptors on oral (Xu et al., 2020) and nasal epithelial cells (Sungnak et al., 2020) are points of entry for SARS-CoV-2. Local viral proliferation in the airway disrupts the ciliated epithelium, induces ciliary dyskinesia and viral movement down the lower respiratory tract epithelium (Chilvers et al., 2001) through the activity of phosphodiesterase, which is a potential therapeutic target (Joskova et al., 2020). Infected bronchial epithelial cells may produce complement (Peters-Hall et al., 2015). In the alveolar epithelium, type I pneumocytes cover approximately 95% of the gas exchange surface area, and the type II pneumocytes cover the remaining area and secrete surfactant in maintaining lung compliance (McElroy and Kasper, 2004). Receptors for SARS-CoV-2 on these cells (Hamming et al., 2004) facilitate infection, increase the production of surfactant and decrease gas exchange in the alveoli (McElroy and Kasper, 2004). SARS-CoV-activated epithelial cells produce cytokines such as IL-6 and IL-8, involved in neutrophil recruitment and reduced lymphocyte activity (Yoshikawa et al., 2009). These cells also undergo apoptosis in COVID-19 (Li et al., 2020). Cell signaling through the pneumocyte surface receptor Mas, associated with the RAAS system (**Figure 4**), prevents angiotensin (Ang) II-induced apoptosis (Gopallawa and Uhal, 2016).

### Macrophages

In the alveolar lumen, macrophages regularly clear debris, surfactant, and microorganisms to maintain lung compliance and protect the epithelium (Curran et al., 2018). The exact interactions of SARS-CoV-2 with macrophages are not clearly known. Macrophages may phagocytose the virus through opsonic mechanisms (e.g. complement, antibodies or surfactant protein-A) or become infected through ACE2 receptor binding interactions (**Figure 2**) (Keidar et al., 2005; Curran et al., 2018). Pathogen- and damage-associated molecular pattern ligands (PAMPs and DAMPs) bind and activate macrophage Toll-like receptors (TLRs) (Zhao and Zhao, 2020). In response to inflammation, macrophages produce the neuropeptide substance P (SP) and the chemokine, monocyte chemoattractant-1 (MCP-1/CCL2), and express the Mas receptor, which attenuates the production of pro-inflammatory cytokines (e.g. IL-6, TNF) upon ligation (Germonpre et al., 1999; Simoes e Silva et al., 2013). Macrophages produce complement factors (e.g. C1q, C1r, C1s, C2, C3, C4, C5) and respond to complement deposited on apoptotic cells or bound to receptors for C3a and C5a (Lubbers et al., 2017). The differentiation of macrophages is regulated, in part, by components of the complement cascade. Specifically, C3a and C5a activate inflammasomes in macrophages, promoting a pro-inflammatory M1-type macrophage whereas the opsonic molecules [C1q, mannose binding lectin (MBL)] promote phagocytosis and reduced inflammation, forming an M2-type macrophage (Bohlson et al., 2014). The neuropeptide SP also promotes the formation of an anti-inflammatory, tissue-reparative M2-type macrophage (Lim et al., 2017). Macrophage polarization states at various stages of infection may play role in viral evasion (Atri et al., 2018).

### Mast Cells

Some viruses are able to activate and infect mast cells, which induces the release of antimicrobial peptides (e.g. cathelicidins) that protect the lung, as well as enzymes (tryptase, chymase) and mediators (e.g. TNF, prostaglandins, histamine), that promote vasodilation and inflammation associated with highly pathogenic infections (Graham et al., 2015; Mollerherm et al., 2016). A functional role of mast cell extracellular traps in viral host defense or inflammation has not been significantly explored (Mollerherm et al., 2016). In the absence of direct viral interaction, mast cells are activated by immunoglobulins (IgE), TLR ligands, complement (C3a, C5a), integrin binding interactions, and thrombin (Theoharides et al., 2011; Graham et al., 2015). Mast cells are additionally activated by tachykinins (SP, neurokinin A, neurokinin B), which bind neurokinin receptors and members of the Mas family of receptors (Kleij and Bienenstock, 2005; Karnik et al., 2017; Green et al., 2019). Mast cells and nerves are in constant contact with each other in both physiologic and pathologic situations and exhibit cross-talk in the brain, lung, and gut (Kleij and Bienenstock, 2005; Traina, 2019). Activated mast cells generate tryptase and serotonin, which induce the release of tachykinins from sensory nerve endings that in turn, activate mast cells (Kleij and Bienenstock, 2005). Mast cells are the major source of heparin throughout the body (Humphries et al., 1999) and produce renin (Theoharides et al., 2011) and angiotensin-converting enzyme (ACE) (Chao et al., 2011) in response to oxidative stress. Mast cells are also a target of approved therapeutics.

### Neurons

The process of respiratory gas exchange sends signals from vagal afferent nerves to respiratory neurons in the brain, which activate muscles in the thorax and abdomen to contract and relax, altering pressure in the lung (Cherniack and Cherniack, 2007). The activation of certain vagal afferent nerves in the respiratory tract can lead to dyspnea and an urge to cough (Mazzone and Undem, 2016). Dry cough is a common feature of COVID-19 (Qin et al., 2020), which could be a result of SARS-CoV-2 binding to neuronal ACE2 (Xiao et al., 2013). Pulmonary unmyelinated sensory neurons (C-fibers) can also be activated by tachykinins or bradykinin to induce cough and the response is enhanced by prostaglandins and histamine (Canning, 2009). Moreover, activated sensory neurons release tachykinins that act on bronchial smooth muscles, the mucosal vasculature, and submucosal glands to promote bronchoconstriction, vascular permeability, edema, and inflammatory cell chemotaxis (Solway and Leff, 1991). The release of SP by intra-adrenal nerve fibers stimulates aldosterone secretion *via* SP binding to neurokinin type 1 receptors (NK1) expressed by aldosterone-producing adrenocortical cells (Wils et al., 2020). The additional finding of SARS-CoV-2 in COVID-19 patient cerebral spinal fluid and the development encephalitis and Guillain-Barré syndrome in some COVID-19 patients identifies a distinct role for neurons in the disease (Ellul et al., 2020).

### Endothelial Cells

The pulmonary capillaries that surround the alveoli consist of a thin layer of endothelial cells to allow rapid exchange of respiratory gases between capillary blood and type I pneumocytes (Gonzales et al., 2015). In examining deceased COVID-19 patient lungs, diffuse alveolar damage and severe endothelial injury associated with intracellular SARS-CoV-2, thrombosis, and signs of intussusceptive angiogenesis were identified (Ackermann et al., 2020). This latter regenerative process of blood vessel splitting in lung injury is a poorly understood process that may contribute to wound repair and restoration of the endothelium (Niethamer et al., 2020). Intussusceptive angiogenesis is also an identified contributor to the progression of cancers and occlusive vascular diseases (Weddell and Imoukhuede, 2018). This process may involve the complement system. Complement factor C1q consists of collagen-like [cC1q] and globular head [gC1q] regions that bind receptors cC1qR and gC1qR, respectively. The activation of both of these receptors on endothelial cells contributes to angiogenesis (Ghebrehiwet et al., 2019). gC1qR also binds factor XII and high molecular weight kininogen in initiating the kallikrein-kinin system and the secondary hemostasis *intrinsic* pathway (Kaira et al., 2020). The latter pathway is additionally triggered by endothelial damage, which produces endothelial collagen that binds platelet glycoprotein VI (GPVI) (Gale, 2011). Endothelial cells can express RAAS pathway enzymes (ACE, ACE2) as wells as receptors that promote [Ang II type 1 receptor (AT_1_R)] and inhibit (Mas, AT_1_R) vasoconstriction, fibrosis, and inflammation (Nehme et al., 2019).

### Platelets

In response to injury, platelets adhere to the endothelium, are activated, and aggregate to form a platelet plug (Reyes Gil, 2019). Platelets are activated by extracellular matrix molecules that bind integrins (Bennett, 2005), complement (Ghebrehiwet et al., 2019), thrombin (Major et al., 2003), and TLR ligands, (Aslam et al., 2006; Koupenova et al., 2014). Influenza ssRNA activates TLR7 in platelets and induces platelet granule release of complement C3, which augments the release of DNA from neutrophils and promotes the formation of platelet–neutrophil aggregates associated with thrombosis (Koupenova et al., 2019). Platelets can also be activated by Ang II and their adhesiveness is down-regulated by ACE2 activity, possibly through Mas activation or through mediators released from Mas-activated endothelial cells (Fraga-Silva et al., 2010). Activated platelets release granules from dense organelles (containing serotonin, ATP, ADP, histamine, thromboxane A2) and alpha organelles (containing factors involved in both promoting and inhibiting fibrinolysis and angiogenesis) (Bhagavan and Ha, 2015; Reyes Gil, 2019).

### Neutrophils

Circulating neutrophils are primed by cytokines (Wright et al., 2010), complement (Fung et al., 2001), and TLR ligands (Wang et al., 2008), which alters their cytoskeletal structure for effective migration across the endothelium, through the pulmonary interstitium, across the epithelium and into alveolar spaces (Potey et al., 2019). In response to thrombin or histamine, endothelial cells rapidly release the most potent neutrophil chemokine, IL-8 (Utgaard et al., 1998). Endothelial cells also produce IL-8, as well as MCP-1, in response to IL-6 complexed to the soluble receptor (sIL-6Ra), which binds the cell surface receptor, gp130 (Romano et al., 1997). In mice, neutrophil trafficking is promoted by IL-6 signaling (JAK/STAT3) through gp130 (Fielding et al., 2008). Neutrophils release neutrophil extracellular traps (NETs), neutrophil elastase, reactive oxygen species (ROS) and proteases to eliminate an invading pathogen, which can also produce additional tissue injury (Potey et al., 2019). Neutrophils shed sIL-6Ra from their cell surface and the IL-6:sIL-6Ra complex stimulates fibroblasts and macrophages to produce MCP-1 involved in the recruitment of monocytes (Kaplanski et al., 2003).

### Monocytes

Increased production of MCP-1 promotes the recruitment of monocytes to the alveoli, where they differentiate into macrophages in response to cytokines, PAMPs and DAMPs in the microenvironment (Huang et al., 2018). A sustained monocyte influx is correlated with the severity of respiratory failure (Rosseau et al., 2000) and may be linked to IL-17 producing T helper cells (Th17) (Jiang et al., 2017).

### Lymphocytes

Circulating lymphocyte numbers decline in severe COVID-19 (Chen G. et al., 2020; Qin et al., 2020). Both CD8+ T cells and CD4+ T cells have elevated levels of the programmed death (PD)-1 receptor in COVID-19 patients, a sign of T cell functional exhaustion (Diao et al., 2020). Compared to healthy controls, COVID-19 patient CD8+ T cells and CD4+ T cells activated *in vitro* produce more IL-17, indicating skewing toward a Th17 phenotype (De Biasi et al., 2020). Because the levels of IL-17 tend to increase with disease severity, an underlying function of Th17 cells has been suggested (Wang J. et al., 2020), despite the lower lymphocyte numbers identified in COVID-19 patients (Chen G. et al., 2020; Qin et al., 2020). Because mast cells and neutrophils are also known to release IL-17 from their extracellular traps (Lin et al., 2011), additional research into the source of IL-17 in COVID-19 patients is needed.

## The RAAS Pathway

The RAAS pathway is a cascade of enzymatic reactions that function in the homeostatic control of extracellular volume, arterial pressure, tissue perfusion, electrolyte balance, and wound healing (Atlas, 2007). A prominent component of the RAAS pathway is located within the juxtaglomerular apparatus. There, the macula densa cells are strategically located in the distal tubule, in a region adjacent to the afferent arteriole, at a point where these elements meet the glomerulus. Sensors in the macula densa cells respond to a low sodium chloride concentration within the lumen of the distal tubule. In response, these cells produce adenosine and ATP, that constrict the afferent arteriole and thereby reduce glomerular perfusion. These cells also produce cyclooxygenase-2 (COX-2), which generates prostaglandins. Juxtaglomerular cells in the afferent glomerular arteriole release pro-renin in response to stimulation by prostaglandins (Kriz, 2004; Atlas, 2007; Peti-Peterdi and Harris, 2010).

Pro-renin is transformed into active renin by proteases, including kallikrein (Biswas et al., 2016). Renin may also be released from mast cells activated by oxidative injury (Theoharides et al., 2011). Liver-produced angiotensinogen is cleaved by renin into Ang I, which is further cleaved by a ubiquitous membrane and soluble ectoprotein, ACE, into Ang II (Igic and Behnia, 2003; Jiang et al., 2014). The binding of Ang II to AT_1_R promotes vasoconstriction; induces the production of aldosterone, which promotes renal tubular sodium reabsorption; has a biphasic effect on sodium reabsorption in the gut (promotes at low concentrations, inhibits at high concentrations) and increases blood pressure (Nehme et al., 2019), which is associated with increased neuronal and immune cell production of the vasodilator, SP (Calvillo et al., 2019). Ang II also binds the type 2 receptor (AT_2_R), which exerts inhibitory actions on AT_1_R cell signals by promoting vasodilation and natriuresis (Atlas, 2007; Nehme et al., 2019). Transformation of Ang II to III and III to IV by aminopeptidases A and N, respectively, activates AT_4_R, which is also known as insulin-regulated aminopeptidase (Goldstein et al., 2017).

The ACE homolog, ACE2, cleaves Ang I into Ang-(1-9) and processes Ang II and Ang-(1-9) into Ang-(1-7), thereby inactivating Ang II. Ang II-mediated signaling is involved in apoptosis, reactive oxygen species production, epithelial-to-mesenchymal transformation (EMT), and alveolar fluid retention (Sriram and Insel, 2020). Thus, ACE2 functions as an endogenous inhibitor of the ACE/Ang II/AT_1_R pathway and opposes the vasoconstrictive, inflammatory, prothrombotic, and fibrotic effects associated with ACE/Ang II/AT_1_R activity (Fraga-Silva et al., 2010; Santos et al., 2018). Consequently, ACE2 and drugs that oppose ACE/Ang II/AT_1_R activity improve the tissue response to injury (Sriram and Insel, 2020). Ang II can also be cleaved by mononuclear leukocyte-derived aspartate decarboxylase (MLDAD), generating angiotensin A (differing from Ang II by one amino acid) that is processed into a newly recognized peptide, alamandine [a decarboxylated form of Ang-(1-7)], by ACE2 (Hrenak et al., 2016; Tetzner et al., 2018). Ang-(1-7) binds Mas and AT_2_R (Karnik et al., 2017). Both Ang-(1-7) and alamandine bind the Mas-related G-protein coupled (MrgD) receptor. These cell signals antagonize inflammation and AT_1_R responses (Hrenak et al., 2016; Karnik et al., 2017; de Carvalho Santuchi et al., 2019) (**Figure 4**).

Moreover, Ang II induces the expression of intercellular cell adhesion molecule-1 (ICAM-1) and vascular cell adhesion molecule-1 (VCAM-1) (Alvarez et al., 2004), P-selectin (Piqueras et al., 2000), and integrins (Kawano et al., 2000; Li et al., 2006) in experimental models. These adhesive interactions promote the recruitment of neutrophils (Piqueras et al., 2000; Alvarez et al., 2004). In *in vitro* assays, cellular ACE2 binds integrin β1 (ITGB1) and integrin α5 (ITGA5), which enhances adhesion and focal adhesion kinase (FAK) signaling, whereas soluble ACE2 inhibits FAK cell signals (Clarke et al., 2012). This may indicate that ectodomain shedding of ACE2 is important in regulating Ang II-induced leukocyte adhesion and recruitment.

In summary, the RAAS pathway is an intricate system that coordinates the activities of many cell types, organ systems and the vasculature to regulate electrolyte balance, blood pressure and cardiovascular function. Dysregulation in this system over an extended period of time promotes renal and cardiovascular diseases (Munoz-Durango et al., 2016). In acute pulmonary infections, the RAAS pathway contributes to the development of acute respiratory distress syndrome (ARDS) and subsequent pulmonary fibrosis (Kuba et al., 2006), which has an estimated incidence rate of 19.5% in COVID-19 patients (Zhu et al., 2020).

## SARS-CoV-2 and RAAS Pathway

ACE2 is expressed in type I and type II pneumocytes, oral and nasal epithelial cells, neurons, and arterial and venous endothelial cells (Hamming et al., 2004; Xiao et al., 2013). In single cell transcriptome data from healthy human lung tissues, ACE2 and TMPRSS2 expression levels were highest in the subsegmental bronchial branches (Lukassen et al., 2020), highlighting likely SARS-CoV-2 binding interactions within the lung. In a murine model, SARS-CoV infection reduced lung function and ACE2 protein levels. A subsequent study by this group assessed a mouse model of acid aspiration-induced lung injury followed by intraperitoneal injection of recombinant SARS-CoV spike-Fc protein compared to a control Fc protein. These data revealed that the spike protein worsened lung injury compared to the control and this response was attenuated by an AT_1_R blocker (ARB) (Kuba et al., 2005). In another model, ACE2 knockout mice exhibited reduced lung function that was improved by the administration of either recombinant ACE2 or an ARB (Imai et al., 2005).

These models demonstrate the potential for interventions that target components of the RAAS pathway to ameliorate acute lung injury. Previous concerns regarding a potential risk of increased SARS-CoV-2 infection in response to these drugs has been alleviated by two large retrospective studies in Italy (Mancia et al., 2020) and the USA (Reynolds et al., 2020). Moreover, in a retrospective review of 42 hospitalized COVID-19 patients with hypertension (median age 64), 17 subjects received ACE inhibitors (ACEi) or ARB therapy and 25 subjects received other antihypertensive drugs. Those treated with ACEi or ARBs had a trend toward less severe disease and tended to have increased T cell counts, reduced viral loads, and lower levels of circulating IL-6 compared to subjects receiving other antihypertensive drugs (Meng et al., 2020). These findings suggest that blocking the RAAS pathway with ACEi or ARBs improves immune responses in COVID-19 patients and indicates a need for randomized controlled trials for further evaluation. In the interim, current clinical guidelines recommend continued use of these therapies in COVID-19 patients with pre-existing conditions such as hypertension (Mehta et al., 2020).

Predicting the possible benefits of a RAAS pathway intervention is complicated by possible ectodomain shedding of ACE2 (Xiao et al., 2014). *In vitro*, ligation of the SARS-CoV S-protein to ACE2 induced the activity of TNF-converting enzyme (TACE) and TACE-dependent shedding of the catalytically active ectodomain of ACE2 (Haga et al., 2008). The functions of soluble ACE2 are not fully known. In patients with heart failure, increased levels of plasma soluble ACE2 correlate with greater disease severity (Epelman et al., 2008). In another study involving patients with acute decompensated heart failure, increased serum soluble ACE2 levels during intensive medical therapy predicted improved outcomes (Shao et al., 2013). These apparently discordant findings indicate that additional study of soluble ACE2 is warranted, particularly prior to using soluble ACE2 as a therapy to block SARS-CoV-2 binding interactions with host cells (Monteil et al., 2020; Zhang H. et al., 2020).

While the effects of ACEi/ARBs and soluble ACE2 in COVID-19 are not fully known, ACE2 is an endogenous inhibitor of the ACE/Ang II/AT_1_R pathway and the downstream vasoconstrictive, inflammatory, prothrombotic, and fibrotic responses (Fraga-Silva et al., 2010; Santos et al., 2018). Possibly, ACE2 dampens Ang II-induced production of interleukin (IL)-6 (via aldosterone) (Luther et al., 2006) and reduces the downstream formation of Th17 cells (Madhur et al., 2010). In COVID-19 patients, plasma IL-6 levels are elevated (Ranucci et al., 2020), prompting the clinical trial investigation of therapeutics that target IL-6 (siltuximab, NCT04329650), its receptor (tocilizumab, NCT04377659), its cell signals [e.g. Janus kinases, JAKs (baricitinib, NCT04373044) and downstream production of IL-17 (secukinumab, NCT04403243)] (Schett et al., 2020). Initial studies in COVID-19 patients with tocilizumab (Klopfenstein et al., 2020; Luo P. et al., 2020; Toniati et al., 2020) and baricitinib (Cantini et al., 2020) have shown promising results. However, tocilizumab use requires caution in patients with hepatic impairment and can induce liver injury in some COVID-19 patients (Muhovic et al., 2020). The potential for additional opportunistic pulmonary infection is also associated with monoclonal antibodies such as tocilizumab. An increased risk of pulmonary infections may also arise in autoimmune patients treated with baricitinib (Khoo et al., 2020) or anti-IL-17 antibodies (ixekizumab) (Mease et al., 2019), suggesting that the use of these drugs requires careful evaluation.

Further exploration of these networks may require targeting Mas, MrgD, or AT_2_R receptors or the use of other pharmacologic interventions, such as direct renin inhibitors or diuretics, which act on several elements of the RAAS (**Table 1**) (Guichard et al., 2013; Byrd et al., 2019). The use of a highly selective NK1 antagonist (aprepitant), which blocks SP binding to NK1 on adrenal zona glomerulosa cells, may also be beneficial in reducing aldosterone levels (Wils et al., 2020) that in turn, promote IL-6 production (Luther et al., 2006). The continued investigation of these pathways will be essential to defining COVID-19 pathophysiology and devising adjunctive therapies to current anti-viral therapies (e.g. remdesivir, ClinicalTrials.gov Identifier: NCT04280705) and immunogenic approaches (**Figures 2** and **3**).

**Table 1:** Medications that Target the RAAS Pathway.

**Drug Class**	**Drug (s)**	**Mechanism of Action**	**Systemic Indication (s)**
Renin Inhibitor	**Aliskiren**	Direct renin inhibitor that specifically reduces plasma renin activity and acts along the RAAS pathway to inhibit the conversion of angiotensinogen to angiotensin I.	Cardiovascular; Endocrine/Metabolic *NCT04432350
Angiotensin Converting Enzyme (ACE) Inhibitors	**Benazepril, Captopril, Enalapril, Enalaprilat, Fosinopril, Lisinopril, Moxipril, Perindopril, Quinapril, Ramipril, Trandolapril**	Inhibits the conversion of Angiotensin I to Angiotensin II providing vasodilatory effects (e.g. increasing bradykinin, prostacyclin) and decreasing aldosterone secretion. May decrease vasoactive kallikreins.	Cardiovascular; Endocrine/Metabolic *NCT04330300
Angiotensin II Receptor Blocker (ARB)	**Azilsartan, Candesartan, Eprosartan, Irbesartan, Losartan, Olmesartan, Telmisartan, Valsartan**	Inhibits angiotensin II selectively by blocking AT_1_R receptor binding, reduces aldosterone and does not act on bradykinin	Cardiovascular; Endocrine/Metabolic *NCT04394117 *NCT04330300
Diuretics: Aldosterone Receptor Antagonists Potassium Sparing	Eplerenone, **Spironolactone** Amiloride, Triamterene	Antagonists that competitively inhibit aldosterone action in the renal distal tubules, affecting electrolyte retention and excretion; anti-fibrotic Decreases intracellular Na^+^ by inhibiting Na^+^ channels in the distal convoluted tubule, decreasing intracellular Na^+^ and impacting Na^+^/K^+^/ATPase function which leads to increased potassium retention	Cardiovascular; Endocrine/Metabolic; Fluid Regulation *NCT04345887
Thiazide Diuretics	**Hydrochlorothiazide**, Chlorthalidone	Acts on electrolyte reabsorption at the distal renal tubule increasing Cl^-^ and Na^+^ excretion	Cardiovascular; Fluid Regulation *NCT04330300
Loop Diuretics	Bumetanide, Ethacrynic Acid, Furosemide, Torsemide	Inhibits reabsorption of Na^+^ and Cl^-^ in the loop of Henle and proximal renal tubule to increase electrolyte elimination; not all drugs in this class work on the distal tubule	Fluid Regulation

*Select ClinicalTrials.gov Identifier for the testing of the **highlighted** listed class of drugs in COVID-19.

## The Complement System

**Figure 5:**
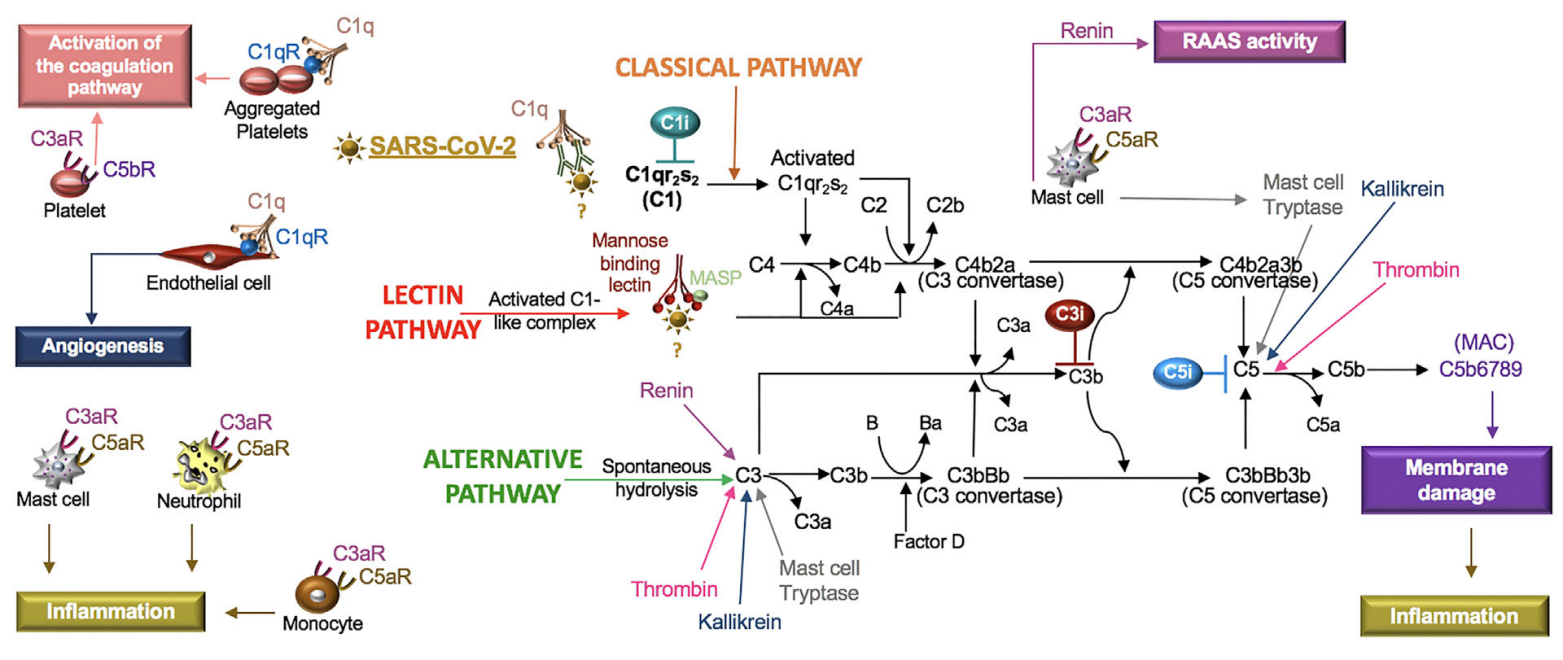
SARS-CoV-2 in the complement system. Classical, lectin, and alternative are the three pathways in the complement system. Complement components C1, C2, C3, and C4 are present in plasma in inactive forms. In the classical pathway, the C1 component, C1q, recognizes apoptotic cells directly or pathogens indirectly through antibody complexes or associations with pentraxins. In the lectin pathway, mannose-binding lectin (MBL) binds the surface of the pathogen. Serine proteases complex with C1q (C1r/C1s) and MBL (MASP: MBL-associated serine protease), which leads to cleavage of C4 to its fragments (C4b and C4a) and the formation of a C3 and C5 convertase. In the alternative pathway, C3 is spontaneously hydrolyzed and through the activity of factor D forms a C3 convertase and subsequently a C5 convertase. Mast cell tryptase, thrombin or kallikrein can also cleave C3 and C5 whereas renin cleaves only C3. Cleavage fragments from these pathways (e.g. C3a, C5a, C5b) activate immune cell subsets to produce inflammation or coagulation. The terminal product of these pathways, C5b6789, is a membrane attack complex (MAC), that creates a pore in cell membranes by displacing phospholipids. The resulting cell lysis induces inflammatory responses. C1q also acts independent of the complement system and binds its receptor (C1qR) on aggregated platelets and endothelial cells in the promotion of coagulation and angiogenesis, respectively. Tissue and organ damage and excessive inflammation in some COVID-19 patients may indicate that SARS-CoV-2 activates the complement cascade. C1, C3, and C5 inhibitors (i) block factor formation in the complement cascade.

The complement system is composed of soluble and cell membrane proteins that regulate the activity of the classical, lectin and alternative pathways (**Figure 5**). The molecules in these pathways act as sensors to tissue damage and pathogens and as effectors to kill microbes and to clear damaged cells (Reis et al., 2019). In the *classical* pathway, C1q recognizes pathogens or apoptotic cells directly or indirectly through antibody complexes, or through associations with pentraxins, such as CRP (Sproston and Ashworth, 2018). In the *lectin* pathway, complement activation is initiated by an interaction involving mannose binding lectin (MBL). Serine proteases [C1r/C1s and MBL-associated serine protease (MASP)] complex with C1q (C1r/C1s) and MBL (MASP). This leads to the cleavage of C4 to its fragments (C4b and C4a) and the formation of a C3 and C5 convertase. In the *alternative* pathway, C3 is spontaneously hydrolyzed and through the activity of factor D, a C3 convertase is formed, which leads to the formation of a C5 convertase (Ricklin and Lambris, 2007). Additionally, C3 and C5 may also be cleaved by mast cell tryptase, thrombin or kallikrein (Ricklin and Lambris, 2007; Ali, 2010).

Activation of any of these pathways results in the insertion of the membrane attack complex (MAC, composed of C5b–9) into targeted cells and generation of active complement fragments such as C3a, C3b, C4a, C4b, and C5a, which bind complement receptors on a various cell types (Reis et al., 2019) (**Figure 5**). C3a and C5a are anaphylatoxins and potent stimulators of neutrophils, monocytes, mast cells and platelets, resulting in the release of mediators and the expression of adhesion receptors (Fung et al., 2001; Ali, 2010). Mast cells at rest produce tissue-type plasminogen activator (t-PA) but in the presence of C5a, mast cells generate plasminogen activator inhibitor (PAI)-1 (Wojta et al., 2002). Moreover, C1q also binds a cell surface receptor, C1qR, on aggregated platelets and endothelial cells, resulting in the activation of the coagulation pathway and angiogenesis, respectively (Ghebrehiwet et al., 2019). In viral infections, such as SARS-CoV, PAMPs and DAMPs bind and activate TLRs (Zhao and Zhao, 2020), which regulate the production and function of complement (Hajishengallis and Lambris, 2016). Thus, the innate immune responses involving the complement system are highly implicated in SARS-CoV-2.

## SARS-CoV-2 and the Complement System

In COVID-19 patients, elevated plasma CRP levels are a prognostic indicator of adverse outcomes. Threshold values of an adverse outcome have been reported as 27 mg/L (Wang G. et al., 2020) and as 41 mg/L (Luo X. et al., 2020). CRP is primarily synthesized by IL-6-dependent hepatic biosynthesis (Sproston and Ashworth, 2018). In a small study of COVID-19 patients, a combination of IL-6 levels > 80 pg/mL and CRP levels > 97 mg/L were highly predictive of the need for mechanical ventilation (Herold et al., 2020). In human skin fibroblasts, IL-6 induces the production of complement factor B and C3 involved in the activation of the alternative pathway (Katz et al., 1989). Because complement participates in various inflammatory skin diseases (Giang et al., 2018), complement may be produced in response to IL-6 in COVID-19 patients, promoting the cutaneous skin disorders characteristic of COVID-19 (Galvan Casas et al., 2020).

Research involving intranasal infection with recombinant mouse-adapted SARS-CoV (MA15) identified C3 fragments in the lungs of mice one day after infection. Additionally, C3-/- mice exposed to this virus manifested reduced neutrophil and monocyte recruitment and less respiratory dysfunction compared to control mice (Gralinski et al., 2018), demonstrating an active role for the alternative pathway in SARS-CoV. The effects of C3 inhibitors (AMY-101 and APL-9) in COVID-19 subjects with ARDS (ClinicalTrials.gov Identifier: NCT04395456 and NCT04402060) are being tested in clinical trials. Mixed reports involving the binding of the SARS-CoV spike protein to MBL (Leth-Larsen et al., 2007; Zhou et al., 2010), indicate that the function of the lectin pathway in SARS-CoV and SARS-CoV-2 requires further study. Lastly, despite increased production of CRP in SARS-CoV-2 (Wang G. et al., 2020), the functions of the classical pathway have not been systematically explored in SARS-CoV or SARS-CoV-2. Clinical investigations with a C1 inhibitor (Conestat alfa, NCT04414631) and a C5 inhibitor (Zilucoplan, NCT04382755) in severe COVID-19 patients are in progress. Monoclonal antibodies against C5 (e.g. eculizumab, ravulizumab) are also available therapeutics that block excessive complement activation.

In cryoinjured mice, ARB treatment was associated with lower systemic and local levels of C1q, decreased fibrosis and increased myofiber regeneration compared to the controls. The response was reversed by topical C1q and the mechanisms were linked to changes in macrophage C1q production (Yabumoto et al., 2015). Excessive activation of macrophages is associated with the pathophysiology of COVID-19 (Verdoni et al., 2020) and may therefore include macrophage C1q production. Blocking Bruton tyrosine kinase (BTK) is a proposed mechanism to suppress macrophage activation (Roschewski et al., 2020) and is currently being tested in clinical trials of COVID-19 subjects (Acalabrutinib, NCT04380688). Blocking BTK may therefore also affect C1q levels. Moreover, in a rat model of Ang II-induced renal damage, increased circulating levels of complement (C1q, C3, C3c, and C5b-9), CRP, and renal TNF were reduced by a direct renin inhibitor (aliskiren) and also by an ARB (losartan) (Shagdarsuren et al., 2005). The exact roles of Ang II and the SARS-CoV-2 receptor, ACE2, in the complement system require further study.

The increased numbers of apoptotic type I and II pneumocytes and endothelial cells in COVID-19 patient lung tissue (Li et al., 2020) are suggestive of a dysregulated host response in the clearance of these cells. This may involve changes in phagocyte cell surface receptors, the activation state of phagocytes, and/or the response of these cells to components of the complement system (Gordon and Pluddemann, 2018). Neutrophils also undergo apoptosis in inflamed tissue but in ARDS, this process is impaired. In assessing the function of peripheral blood neutrophils from ARDS patients, neutrophils were activated *in vitro* and produced more NETs and exhibited increased viability compared to healthy control neutrophils. In addition, human monocyte-derived macrophages from the ARDS patients were unable to effectively phagocytose apoptotic neutrophils. However, in the presence of metformin, a 5’ AMP-activated protein kinase (AMPK) activator, the response was improved (Gregoire et al., 2018). Complement component C1q also induces AMPK activation in macrophages (Galvan et al., 2014) and similar to ARDS patients, COVID-19 patient serum exhibits increased NET activity (Zuo et al., 2020). Thus, SARS-CoV-2 may alter the activity or production of C1q, its receptor, the C1 proteases (C1r, C1S), and/or the regulatory crosstalk known to occur between complement and the various TLR ligands (e.g. dsDNA and ssRNA) released from NETs and apoptotic cells (Hajishengallis and Lambris, 2016).

Mast cell-produced tryptase contributes to the cleavage of C3 and C5 (Ali, 2010). Mast cell-produced renin cleaves C3 but not C5 (Bekassy et al., 2018). Because ligands from C3 and C5 cleavage (C3a, C5a) activate mast cells (Ali, 2010) and mast cells may additionally produce ACE (Chao et al., 2011), persistent cross-talk between the complement system and mast cells likely maintains homeostasis in the RAAS and complement pathways. Mast cells also express Mas-related G protein coupled receptor X2 (MRGPRX2) (Ali, 2016). The Ang-(1-7) receptors (Mas, MrgD) and MRGPRX2 are members of a family of ~40 orphan receptors that exhibit ligand promiscuity with AT_1_R and AT_2_R in the regulation of the RAAS (Karnik et al., 2017). Moreover, Ang III activates Mas and MRGPRX2 (Gembardt et al., 2008), indicating that Ang III may compete with the ACE2 product, Ang-(1-7), in binding to its receptors (Mas, MrgD, and AT_2_R) (**Figure 4**).

The mast cell receptor MRGPRX2 also binds the neuropeptide SP and in response, mast cells produce chemokines (Green et al., 2019). SP, a tachykinin, binds NK1, is released from immune cells and neurons, and enhances inflammatory processes in the lung, gut, and skin (Johnson et al., 2016), which are common sites of inflammation and sources of symptoms in COVID-19 patients (Galvan Casas et al., 2020; Wong et al., 2020). SP and the complement fragments (C3a, C5a) similarly activate mast cells *via* distinct pathways (el-Lati et al., 1994). SP also acts synergistically with C5a in the recruitment and activation of neutrophils (Perianin et al., 1989). Regulatory cell signals generated by complement fragments and the ligands to Mas-related receptors in mast cells, neutrophils and additional immune subsets may be important to the recruitment of neutrophils and the pathogenesis of various COVID-19 inflammatory disorders.

## The Coagulation Pathway

The hemostatic system is divided into three phasic processes. In primary hemostasis, activated platelets aggregate to form a platelet plug. In secondary hemostasis, activated coagulation factors on the surface of the endothelium and platelets form a fibrin mesh that stabilizes the plug, forming a fibrin clot. These processes are balanced by tertiary hemostasis, which activates fibrinolysis for the dissolution of the clot. During primary hemostasis, platelets adhere to von Willebrand factor released by the injured endothelium. Their activation is induced by various PAMPs and DAMPs, which encourages platelet aggregation and the formation of a necessary physical platform for the activation of the coagulation cascade (Reyes Gil, 2019).

Secondary hemostasis involves a cascade of serine proteases that are subdivided into an intrinsic pathway (surface-contact factors) and an extrinsic pathway (tissue factor initiated). The *intrinsic* pathway commences after endothelial damage with the release of endothelial collagen. This activates factor XII to factor XIIa, which acts as a catalyst to activate factor XI to factor XIa. Factor XIa converts factor IX to factor IXa and factor IXa converts factor X to factor Xa, which is a point of convergence in the two pathways. In the *extrinsic* pathway, local tissue injury releases tissue factor into the blood. Tissue factor activates factor VII to factor VIIa and the complex of tissue factor and factor VIIa activates factor X and factor IX. The resulting factor Xa is incorporated into a prothrombinase complex (composed of factor Xa: factor Va: Ca^2+^: platelet phospholipid) that converts factor II (pro-thrombin) to factor IIa (thrombin) (Gale, 2011). This initiates the coagulation cascade and the accumulation of thrombin, which exhibits proteolytic functions, acting upon substrates such as fibrinogen and factor XIII (fibrin stabilizing factor), resulting in the production of fibrinopeptides and active factor XIII (FXIIIa), respectively (Stassen et al., 2004; Huntington, 2005). Concomitantly, thrombin induces the activation of mast cells that produce molecules that promote (IL-6) and inhibit (heparin) coagulation (Theoharides et al., 2011). The fibrinopeptides spontaneously polymerize to form fibrin, which is covalently cross-linked by FXIIIa to form a stable nascent fibrin clot (Huntington, 2005).

**Figure 6:**
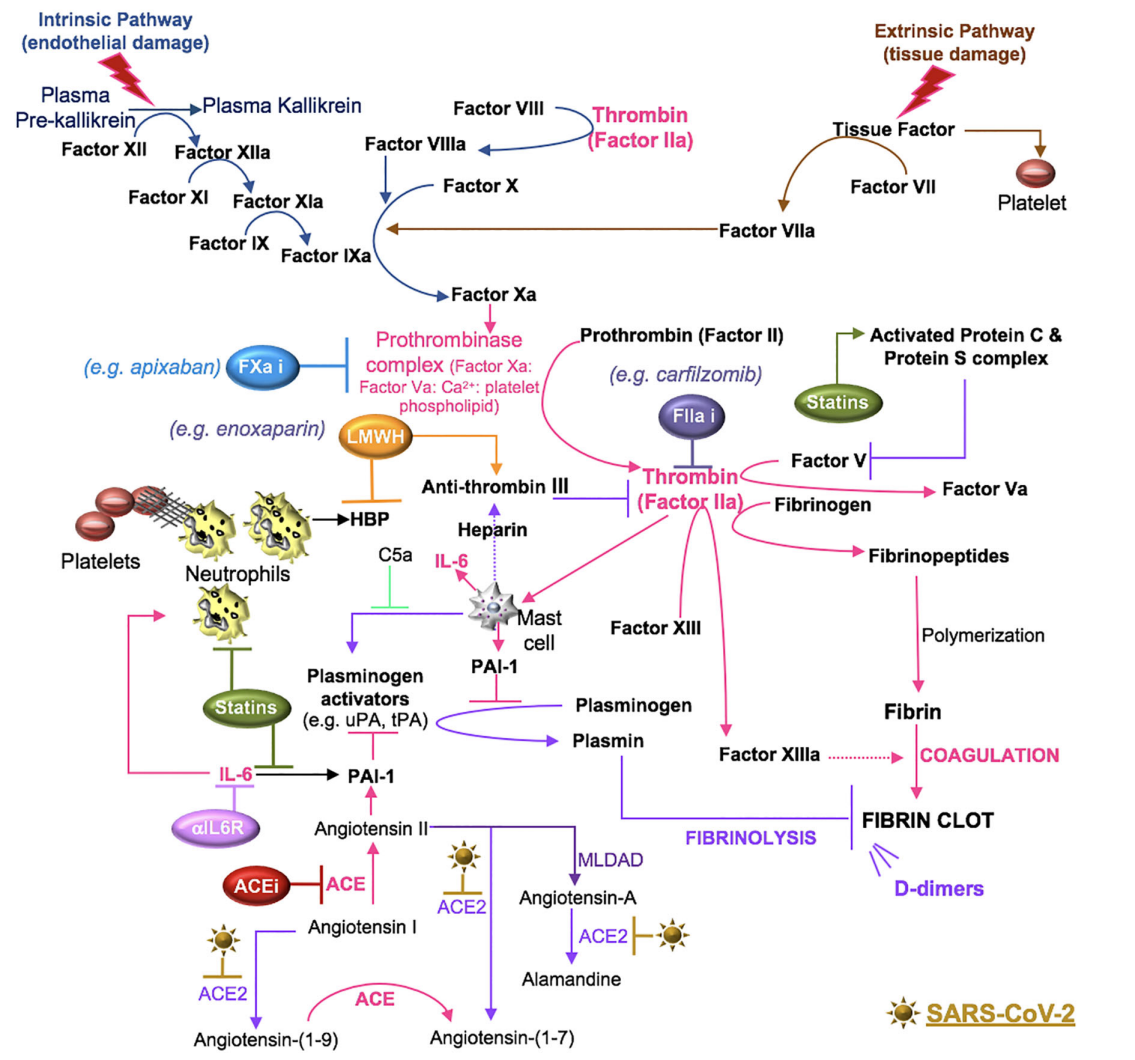
SARS-CoV-2 in coagulation. Primary hemostasis controls platelet aggregation and secondary hemostasis promotes fibrin formation through the clotting cascade, the latter involving intrinsic and extrinsic pathways. In the intrinsic pathway, damage-induced release of endothelial collagen activates factor XII (factor XIIa), which is a reaction that is also involved in the initiation of plasma kallikrein. Factor XIIa acts as a catalyst to activate factor XI to factor XIa. Factor XIa activates factor IX to factor IXa and the latter, acting with factor VIIIa as a cofactor, activates factor X to factor Xa. Tissue injury releases tissue factor into the blood, which activates platelets to induce neutrophil extracellular trap (NET) formation. The components of NETs reciprocally activate platelets and their aggregation. In the extrinsic pathway, tissue factor activates factor VII (factor VIIa), which activates factor X (factor Xa). The common coagulation pathway commences at factor X, with factor Xa, factor Va, calcium and platelet phospholipids forming the prothrombinase complex, which activates prothrombin (factor II) to thrombin (factor IIa). Thrombin cleaves factor V, VIII, factor XIII and fibrinogen. Polymerization of the formed fibrinopeptides produces fibrin, which is covalently cross-linked by FXIIIa to form a stable nascent fibrin clot. These processes can be antagonized by the heparin-dependent activity of antithrombin and plasmin degradation of fibrin into soluble fibrin degradation products (e.g. D-dimer). Plasmin formation is reduced by angiotensin II- or IL-6-induced plasminogen activator inhibitor (PAI)-1, which antagonizes the activity of urokinase plasminogen activator (uPA) and tissue plasminogen activator (tPA). SARS-CoV-2-induced degradation of ACE2 reduces ACE2 cleavage of angiotensin I and II and the anti-inflammatory effects of the ACE2 fragment [Ang-(1-7)], suggesting that inhibitors of RAAS [e.g. ACE inhibitor (ACEi)] may counter the effects of SARS-CoV-2. Mast cells activated by thrombin or complement (C5a) also contribute to coagulation through the preferential production of IL-6 and PAI-1. Drugs that inhibit IL-6 signals (e.g. anti-IL-6 receptor antibodies) may inhibit IL-6-induced PAI-1 production and IL-6 recruitment of neutrophils. Statins also inhibit IL-6 production, the activity of neutrophils and the functions of activated protein C. The use of inhibitors (i) to the clotting cascade such as factor Xa (e.g. apixaban) and factor IIa (e.g. carfilzomib) may impede SARS-CoV-2-induced coagulation responses. Lastly, low molecular weight heparin (LMWH) therapy may promote the effects of anti-thrombin and inhibit the functions of the alarmin, heparin binding protein (HBP), which binds glycosaminoglycan moieties of cell surface proteoglycans and promotes endothelial permeability.

Tertiary hemostasis involves clot dissolution and the initiation of wound remodeling (Reyes Gil, 2019). Plasmin is generated by plasminogen activators, such as urokinase plasminogen activator (uPA) and tissue plasminogen activator (tPA), which cleave plasminogen into the proteolytically active plasmin enzyme (Stassen et al., 2004). This process of fibrinolysis is controlled by plasminogen activator inhibitors (PAI-1 and PAI-2) and plasmin inhibitors (α2-antiplasmin and α2-macroglobulin) (Stassen et al., 2004) (**Figure 6**).

## SARS-CoV-2 and Coagulation

Elevated circulating plasminogen levels may contribute to SARS-CoV-2 susceptibility and worse clinical outcomes in patients with hypertension, diabetes, coronary heart disease, and chronic kidney disease (Ji et al., 2020). Because Ang II induces the production of PAI-1 in endothelial cells (Vaughan et al., 1995), SARS-CoV-2 may indirectly regulate PAI-1 *via* ACE2 down-regulation. In a rat model of abdominal vena cava thrombosis, decreased ACE2 activity is associated with thrombus formation whereas pharmacological (e.g. xanthenone) activation of ACE2 attenuates platelet vessel attachment and thrombus formation (Fraga-Silva et al., 2010), highlighting a potential regulatory role of ACE2 in coagulation.

A retrospective study of hospitalized COVID-19 patients indicates a potential benefit of anticoagulation therapy, particularly in mechanically-ventilated patients (N = 395, 29% mortality), compared to patients that did not receive anticoagulation therapy (63% mortality). Consequently, algorithms are evolving regarding diagnostic markers (IL-6, D-dimer levels, pro-thrombin time, fibrinogen, chest vessel enlargement on computerized tomography) to identify and implement best practice approaches to prophylactic and therapeutic anticoagulation therapy (Oudkerk et al., 2020). Elevated D-dimer levels in COVID-19 patients are common and indicative of coagulation, secondary hyperfibrinolysis, and a possible increased risk of venous thromboembolism (Cui et al., 2020; Tang et al., 2020b; Yin S. et al., 2020). In COVID-19 patients with D-dimer levels more than six-fold over the upper limit of normal, low molecular weight heparin (LMWH) therapy may be associated with better prognosis (Tang et al., 2020a). In another study of COVID-19 patients with elevated D-dimer levels, increased fibrinogen levels were also identified and associated with increased IL-6 levels (Ranucci et al., 2020). Because IL-6 also induces PAI-1 production (Dong et al., 2005; Rega et al., 2005), which promotes a prothrombotic state, this inflammatory cytokine is a therapeutic target in the coagulation pathway (**Figure 6**).

Moreover, circulating levels of IL-6 induce the recruitment of neutrophils to the lung (Suwa et al., 2001). Activated neutrophils release the alarmin, heparin binding protein (HBP), which binds glycosaminoglycan moieties of cell surface proteoglycans on the endothelium, promoting endothelial permeability; this pro-inflammatory response can be attenuated by LMWH (Fisher and Linder, 2017). The interactions between activated neutrophils and platelets also promote coagulation (Zucoloto and Jenne, 2019). HMG CoA reductase inhibitors (statins) reduce the activation of neutrophils (Fisher and Linder, 2017), promote fibrinolysis through increased activated protein C (Undas et al., 2014) and inhibit IL-6-induced PAI-1 production (Dong et al., 2005). For all these reasons, statins may have a therapeutic effect in COVID-19-induced thrombotic responses.

C5a activation of mast cells stimulates production of plasminogen activator inhibitor (PAI)-1 and antagonizes t-PA production (Wojta et al., 2002), suggesting that additional mast cell activators, such as thrombin (Theoharides et al., 2011) and SP (Hermans et al., 2018), may have similar effects. SP also induces monocyte tissue factor expression (Khan et al., 2012) and promotes platelet clot formation (Azma et al., 2009). Understanding SP binding interactions with NK1 (Johnson et al., 2016) and Mas-related receptors (Green et al., 2019) on monocytes, mast cells and platelets may therefore be important hemostatic regulators in COVID-19 patients.

Additional approaches to modulate the coagulation cascade may include direct thrombin inhibitors, factor Xa inhibitors, and heparin, and these are all being assessed in clinical trials (**Table 2**) (Stassen et al., 2004; Padmanabhan, 2014; Solari and Varacallo, 2020). Clarifying the roles of these agents in the context of COVID-19 pathophysiology is needed to better understand the associated thrombotic complications that arise in the clinical course of infection. This research may also shed light on complement-mediated microvascular injury and thrombosis in COVID-19 patients (Magro et al., 2020).

**Table 2:** Medications that Target the Clotting Cascade.

**Class**	**Drug (s)**	**Mechanism**	**Indications**
Direct Thrombin Inhibitors	**Argatroban**, **Bivalirudin**, Dabigatran, Desirudin,	Inhibits coagulation through inhibition of free and fibrin bound thrombin, clotting cascade, and platelet aggregation	Cardiovascular, Vascular; prophylaxis *NCT04406389 *NCT04445935
Vitamin K Antagonists	Warfarin, Acenocoumarol	Coumarin and coumarin derivatives inhibit vitamin K epoxide reductase which inhibits the process of Vitamin K dependent clotting factor synthesis	Cardiovascular, Vascular; prophylaxis
Factor Xa Inhibitors	Apixaban, Betrixaban, Edoxaban, **Fondaparinux**, **Rivaroxaban**	Selective, reversible inhibitors of free and bound factor Xa inhibits platelet activation and coagulation	Cardiovascular, Vascular *NCT04406389 *NCT04416048
Low Molecular Weight Heparin (LMWH)	**Dalteparin**, **Enoxaparin**, Tinzaparin, Nadroparin	Inhibits factor Xa by activating antithrombin III to prevent clotting	Cardiovascular, Vascular; prophylaxis *NCT04412304 *NCT04373707
Heparin and Heparinoids	**Heparin**, Danaparoid sodium	Binds to antithrombin III and prevents the conversion of prothrombin to thrombin and fibrin to fibrinogen by inactivation of coagulation factors (Xa, thrombin)	Anticoagulation *NCT04397510
Glycoprotein IIb/IIIa Inhibitors	Abciximab, Eptifibatide, **Tirofiban**	Binds to glycoprotein IIb/IIIa receptor and blocks platelet aggregation	Cardiovascular *NCT04368377
ADP Induced Aggregation Inhibitors	Cangrelor, **Clopidogrel, Prasugrel**, Ticagrelor, Ticlopidine	P2Y_12_ platelet receptor inhibitors prevent ADP-induced platelet activation and aggregation	Cardiovascular, Vascular; Surgical; prophylaxis *NCT04333407 *NCT04445623
Protease-Activated Receptor (PAR-1) Antagonists	Vorapaxar	Reversible PAR-1 inhibitor blocks thrombin and thrombin receptor agonist peptide platelet aggregation	Cardiovascular, Vascular; prophylaxis
Tissue Plasminogen Activators (TPA)	**Alteplase**, Reteplase, Streptokinase, Tenecteplase, Urokinase, **Defibrotide**	Converts plasminogen to plasmin which cleaves fibrin clots	Cardiovascular; Vascular *NCT04357730 *NCT04348383
Activated Protein C	Ceprotin	Reduces thrombin formation through the inactivation of factor Va and factor VIIIa and inhibits PAF-1	Vascular; prophylaxiss
Kallikrein Inhibitor	**Lanadelumab**	Binds kallikrein to reduce bradykinin production	Fluid Regulation *NCT04422509

DVT, deep venous thrombosis. Common pharmaceutical suffixes can be decoded as follows: -an, antagonist; -ib, inhibitor; –mab, monoclonal antibody *Select ClinicalTrials.gov Identifier for the testing of the **highlighted** listed class of drugs in COVID-19.

## The Kallikrein-Kinin System

The kallikrein-kinin system is an enzymatic cascade of molecules whose functions are interlaced with the activation of the coagulation and RAAS pathways and associated with vascular permeability and inflammation (Nokkari et al., 2018). The process begins with prekallikrein sourced from tissue and plasma. Tissue prekallikrein (KLK1, true tissue kallikrein) is found in arteries and veins; various organs including heart, pituitary and adrenal glands; and immune cell subsets including neutrophils (Rhaleb et al., 2011; Lizama et al., 2015). Release of proteolytic enzymes in damaged tissue activates tissue prekallikrein to form kallikrein, which catalyzes the formation of kallidin from low molecular weight kininogen (Nokkari et al., 2018). The enzymatic interaction of kallidin with an aminopeptidase generates bradykinin, which is also a derivative of plasma kallikrein (KLKB1, Fletcher factor) (Rhaleb et al., 2011; Nokkari et al., 2018). Plasma prekallikrein is mainly produced in the liver, activated by Hageman factor (factor XII), and preferentially releases bradykinin from high molecular weight kininogen (Nokkari et al., 2018). Kallidin and bradykinin can be further processed by carboxypeptidase-M/kininase I into des-Arg^10^-kallidin and des-Arg^9^-bradykinin, respectively, and both of these peptides activate the bradykinin 1 receptor (B1R) (Kakoki and Smithies, 2009). Kallidin and bradykinin activate bradykinin 2 receptor (B2R) and both can be degraded by angiotensin-converting enzyme (ACE, kininase II) into inactive peptides (Kakoki and Smithies, 2009; Nokkari et al., 2018).

The activation of B1R and B2R receptors induces a calcium flux that promotes nitric oxide (NO) production and the activation of phospholipase A_2_ (PLA_2_) enzymes, which liberates arachidonic acid from cell membrane phospholipids (Park et al., 2006; Kakoki and Smithies, 2009). Constitutive cyclooxygenases-1 (COX-1) and inducible cyclooxygenases-2 (COX-2), that may be induced by B2R NF-κB activation (Chen et al., 2004), oxidize arachidonic acid to form prostaglandins (Park et al., 2006). Prostaglandins promote vasodilation and can be released from activated neutrophils (e.g. TNF or IL-1ß activation) as a compensatory mediator to down-regulate inflammation (Wright et al., 2010).

**Figure 7:**
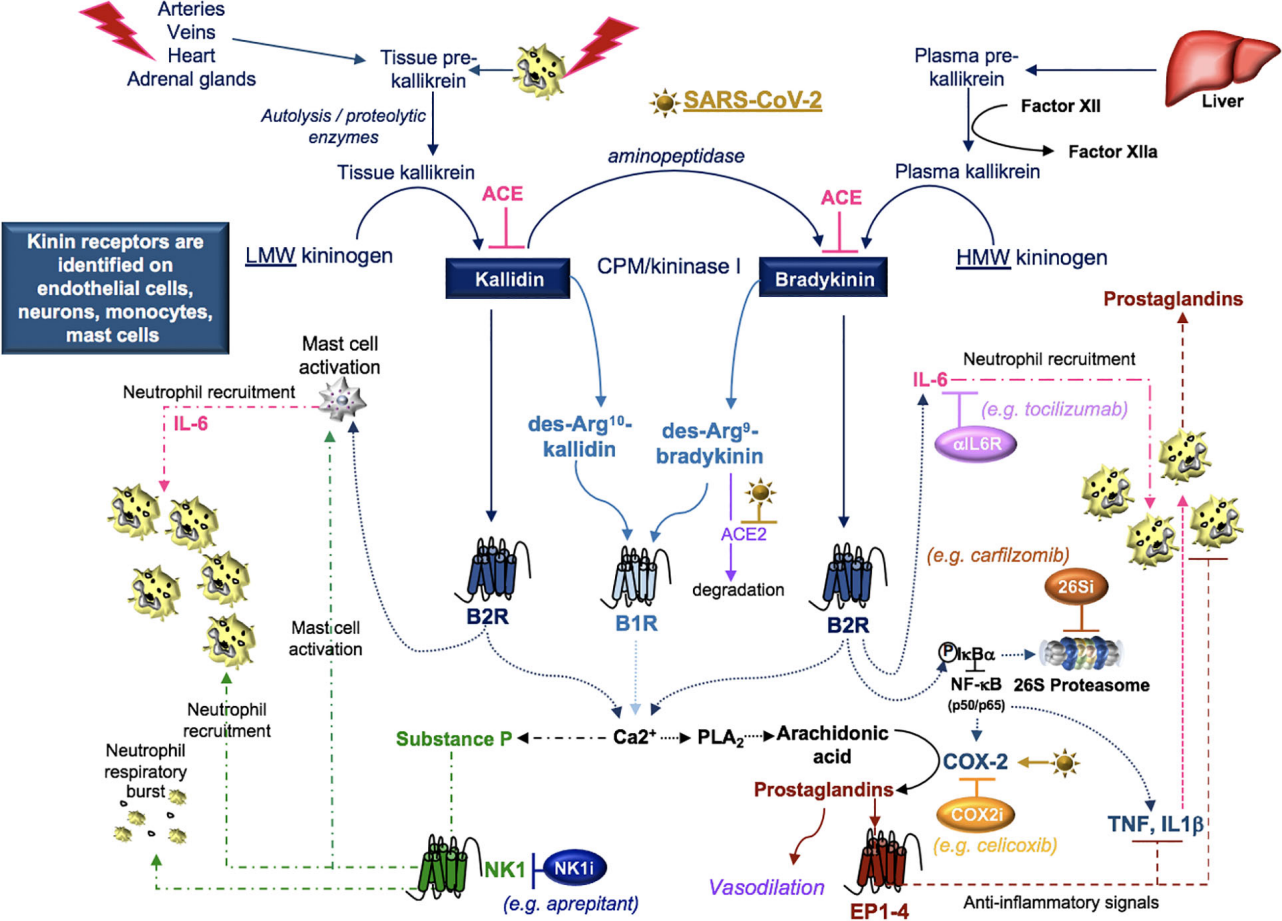
SARS-CoV-2 and kinins. Tissue prekallikrein released from neutrophils and additional cell types is cleaved by cell surface proteases into tissue kallikrein, which promotes the transformation of low molecular weight (LMW) kininogen into kallidin. An aminopeptidase cleaves kallidin and generates bradykinin, which is a product of factor IIa-activated plasma kallikrein and high molecular weight (HMW) kininogen. Kallidin and bradykinin can be cleaved by ACE (kininase II) to form inactive peptides or carboxypeptidase-M (CPM/kininase I), which forms bradykinin 1 receptor (B1R) ligands des-Arg10-kallidin and des-Arg9-bradykinin. ACE2 is the SARS-CoV-2 receptor, suggesting that ACE2 degradation of des-Arg9-bradykinin may be impaired in COVID-19 patients. Both kallidin and bradykinin activate the bradykinin receptor, B2R, which induces the activation of mast cells, the phosphorylation/degradation of IkBα through the 26S proteasome and the release of NF-kB transcription factors involved in the production of cytokines and the prostaglandin-generating enzyme COX-2. Proteasome inhibitors (26Si) antagonize NF-kB activation. B1R and B2R induce a calcium flux that promotes substance P production and the activation of phospholipase A_2_ (PLA_2_) enzymes. PLA_2_ liberates arachidonic acid for COX-2-induced production of prostaglandins which can be blocked with COX-2 inhibitors (COX2i). Prostaglandins induce anti-inflammatory signals. Substance P and bradykinin-induced IL-6 recruit neutrophils into tissues. Substance P also promotes the degranulation of mast cells and neutrophils. Neurokinin-1 (NK1) receptor antagonist (NKi) may block neutrophil recruitment and respiratory burst activity.

Moreover, activation of B1R and B2R on neurons induces a calcium flux that releases SP (Thornton et al., 2010). Tachykinins (SP, neurokinin- A and B, neuropeptide- K and γ) bind neurokinin receptors (NK1, NK2, NK3) where SP exhibits a high affinity for NK1 on nerve cells localized in bronchial vessels, and on epithelial cells, submucosal glands and the vascular endothelium (O’Connor et al., 2004). Consequently, SP contributes to the regulation of cardiovascular and respiratory function, emetic reflux, and the recruitment and activation of leukocytes in response to pathogens, allergens, and self-antigens (O’Connor et al., 2004; Thornton et al., 2010; Mashaghi et al., 2016) (**Figure 7**).

## SARS-CoV-2 and the Kallikrein-Kinin System

Disruption of the kallikrein-kinin system is identified in cardiovascular diseases, chronic kidney diseases, and Alzheimer disease (Kayashima et al., 2012; Ji et al., 2019) and each of these conditions can increase the susceptibility to SARS-CoV-2 (Ji et al., 2020; Kuo et al., 2020). SARS-CoV-induced loss of ACE2 (Kuba et al., 2005) reduces ACE2-dependent degradation of des-Arg^9^-bradykinin (Sodhi et al., 2018) and activates this pathway (**Figure 7**). Further, in a mouse model of acute lung infection, neutrophil influx in response to endotoxin was associated with reduced ACE2 lung protein production. This response was attenuated by a B1R antagonist (Sodhi et al., 2018), suggesting that a loss of ACE2 in the lung enhances neutrophil recruitment through the production of bradykinin and the des-Arg^9^-bradykinin peptide. In additional studies, neutrophil influx was amplified by B1R- and B2R-induced production of prostaglandins and SP, which promote vasodilation and neutrophil recruitment (O’Connor et al., 2004; Thornton et al., 2010; Nokkari et al., 2018). In COVID-19 patients, the neutrophil-to-lymphocyte ratio increases with disease severity (Chen G. et al., 2020; Qin et al., 2020). This may indicate that blocking kallikrein (ClinicalTrials.gov Identifier: NCT04422509) or B2R (van de Veerdonk et al., 2020) may reduce neutrophilia in COVID-19 patients.

Cellular responses to B1R and B2R activation are implicated in several autoimmune disorders that can manifest in the skin and central nervous system (Dutra, 2017). Increasingly, various atypical skin lesions (Jimenez-Cauhe et al., 2020; Sachdeva et al., 2020) and central nervous disorders (Yin R. et al., 2020), including demyelinating lesions (Zanin et al., 2020), are identified in COVID-19 patients. Whether SAR-CoV-2 contributes to the promotion of autoimmunity in COVID-19 patients through kallikrein-kinin system activation is not known.

An important target of B2R cell signaling is NF-κB (Chen et al., 2004), which is activated by the phosphorylation and targeted proteasomal degradation of inhibitors of NF-κB (e.g. IκBα) (Liu et al., 2017). In two different murine models involving the murine hepatitis virus strain (MHV), conflicting results with the proteasome inhibitor (PS-341/bortezomib/Velcade) revealed improved survival in one model involving the MHV-1 strain (Ma et al., 2010) and increased infection and mortality in the other model involving the MHV-A59 strain (Raaben et al., 2010a). These differences may, in part, be due to differences in the virus (MHV-1 (pneumotropic) versus MHV-A59 (neurotropic)), mouse strains (A/J versus C57BL/6), drug concentrations (0.25 mg/kg versus 1 mg/kg), and the route of administration (subcutaneous versus intraperitoneal), respectively. In both studies, drug-induced inhibition of viral replication *in vitro* was noted and has been further supported in studies identifying the importance of the ubiquitin proteasome system in viral entry, RNA synthesis and mRNA translation (Raaben et al., 2010b). Bortezomib is commonly used in the treatment of multiple myeloma and in particular patient subsets, severe pulmonary toxicity has been reported (Li et al., 2016). The effects of proteasome inhibition in COVID-19 are not clear; however, the extensive pro-inflammatory response in severe COVID-19 patients (Chen G. et al., 2020; Qin et al., 2020) suggests that drugs targeting NF-κB (**Table 3**) (Baeuerle and Baichwal, 1997; Miller et al., 2010) could be of benefit in dampening excessive cytokine responses in some COVID-19 patients.

**Table 3:** Medications that Target the Immune Response.

**Targets**	**Class**	**Drugs**	**Mechanism**	**Indications**
Nitric oxide, NF-*k*B, proteasome, cytokines	HMG-CoA reductase inhibitors (statins)	**Simvastatin, Atorvastatin**, Cerivastatin, Fluvastatin, Lovastatin, Pitavastatin, Pravastatin, Rosuvastatin	Inhibits RLS in cholesterol synthesis and decreases circulating lipids while also decreasing C-reactive protein levels and reducing inflammation	Cardiovascular; Hepatic; Metabolic *NCT04348695 *NCT04380402
NF-κB pathway	Anti-TNF agents	Adalimumab, Certolizumab pegol, Etanercept, Golumumab, **Infliximab**	Inhibits TNF cell signals	Rheumatic *NCT04425538
NF-κB pathway	Protease antagonists	Bortezomib, Carfilzomib, Ixazomib	Inhibits proteasomes responsible for cell homeostasis and promotes proapoptotic activity	Cancer
NF-κB pathway	Interleukins	Rilonacept (IL-1) **Anakinra (IL-1R)** **Canakinumab (IL-1β)** **Tocilizumab (IL-6R)** **Sarilumab (IL-6R)** **Siltuximab (IL-6)** Brodalumab (IL-17) Ixekizumab (IL-17) **Secukinumab (IL-17)**	Cytokines involved in immunoregulatory and inflammatory processes	Cancer *NCT04443881 *NCT04362813 *NCT04377659 *NCT04315298 *NCT04329650 *NCT04403243
Pattern-recognition receptors, toll-like receptor 3	Interferons	**Interferon Alfa-2b**, Interferon Alfa-n3, **Interferon Beta-1a**, Interferon Gamma-1b, PEG interferon Alfa- 2a, PEG interferon Alfa-2b, PEG interferon Beta-1a	Cytokines which have antiviral response activity and immunomodulatory capacity	CNS; Cancer *NCT04379518 *NCT04449380
Stress	Systemic glucocorticoids	Betamethasone, **Budesonide**, Cortisone, Cosyntropin (ACTH), Deflazacort, **Dexamethasone**, Fludrocortisone, **Hydrocortisone, Methylprednisolone**, Prednisolone, **Prednisone**, Triamcinolone	Anti-inflammatory actions include suppression of neutrophil function and inhibition of prostaglandin synthesis	Allergy; Autoimmune; Dermatologic; Respiratory; Cancer; Gastrointestinal; Hematologic; Ophthalmic; Rheumatic; Renal *NCT04361474 *NCT04325061 *NCT04359511 *NCT04263402 *NCT04344288
Cytokines/Mast Cell	Antihistamines 1^st^ generation 2^nd^ generation	Diphenhydramine, Pyrilamine, Doxylamine, Brompheniramine, Dexbrompheniramine, Carbinoxamine, Chlorpeniramine, Clemastine, Dexchlorpheniramine, Dimenhydrinate, Meclizine, Promethazine, Triprolidine Acrivastine, Certizine, Desloratidine, Fexofenadine, Levocertirizine, Loratadine	Competitive for H1 receptors on effector cells (gastrointestinal, respiratory, blood vessels); sedating Competitive for H1 receptors on effector cells (gastrointestinal, respiratory, blood vessels); does not cross BBB, less sedating	Allergic Reactions
Cytokines/Mast Cell	Histamine 2 antagonists	**Famotidine**, Cimetidine, Nizantidine, Ranitidine	Competitively inhibits the histamine H2 receptor on the parietal cells to decrease gastric acid secretion	Mast cell disease, Gastro-esophageal reflux *NCT04370262
Cytokines/Mast Cell	Leukotriene receptor antagonists	**Montelukast**, Zarfilukast	Selective antagonist of cysteinyl leukotrienes to reduce airway inflammation and edema	Asthma *NCT04389411
Cytokines/Mast Cell	Mast cell stabilizers	Cromolyn, Ketotifen	Inhibits the release of histamine, leukotrienes, and mediators	Systemic mastocytosis; Asthma
JAKs	Janus Associated Kinase	**Baricitinib**, Fedratinib, **Ruxolitinib, Tofacitinib**, Upadacitinib	Inhibits JAK-induced cytokine and growth factor expression and signaling activity, including STAT activation	Rheumatoid Arthritis *NCT04421027 *NCT04377620 *NCT04412252
Substance P	Neurokinin-1 receptor antagonist	Aprepitant, Fosaprepitant, Netupitant, Rolapitant	Selectively and competitively inhibits substance P binding to neruokinin-1 and downstream signals	Antiemetic
COX-2	Cyclooxygenase (COX)-II Selective	Celecoxib, Parecoxib, Etoricoxib	Selective inhibition of COX-2 decreases prostaglandin synthesis; anti-inflammatory; antipyretic; analgesic	Pain; Arthritis

Shown are medications that suppress adaptive and innate immune responses.

CNS, central nervous system; HMG-CoA reductase, 3-hydroxy-3-methylglutaryl coenzyme A reductase; RLS, rate limiting step; PEG, polyethylene glycol; NFκB, nuclear factor kappa B; TNF, tumor necrosis factor. Infix -zu denotes a humanized monoclonal antibody. Suffix –mab indicates a monoclonal antibody. Suffix -ib denotes a small molecule inhibitor. *Select ClinicalTrials.gov Identifier for the testing of the **highlighted** listed class of drugs in COVID-19.

B2R also signals through NF-κB to induce cytokine production and to activate COX-2, promoting prostaglandin production (Chen et al., 2004). The SARS-CoV components, S-protein (Liu et al., 2007) and N-protein (Yan et al., 2006), are implicated in the activation of COX-2 *via* calcium dependent and independent signaling. Inhibition of COX-2 *via* siRNA or the NS-398 COX-2 inhibitor revealed that COX-2 is required for replication of the MHV-A59 strain *in vitro* (Raaben et al., 2007). In a murine model of influenza A/H5N1, intraperitoneal treatment with the antiviral neuraminidase zanamivir improved viability that was further enhanced with the addition of the COX-2 inhibitor celecoxib (Zheng et al., 2008). In COX-2 deficient mice infected with influenza A/Hong Kong/8/68 (H3N2), the COX-2-deficient mice exhibited enhanced survival compared to wild-type controls (Carey et al., 2005), highlighting the potential benefits of COX-2 inhibition in viral infection.

Non-steroidal anti-inflammatory drugs (NSAIDs), such as ibuprofen, antagonize COX enzymes. In a rat model of streptozotocin-induced diabetes, oral gavage with ibuprofen reduced the production of Ang II and ACE but induced the production of ACE2 in cardiac tissue (Qiao et al., 2015). In murine bone marrow-derived dendritic cells and human peripheral blood monocytes, B1R and B2R agonists or an ACE inhibitor (captopril) blocked recombinant interferon-α- or TLR-induced type I interferon responses. In the additional presence of an NSAID (indomethacin), the type I interferon response was restored (Seliga et al., 2018). Because SARS-CoV is able to delay TLR-induced type I interferon responses (Kindler et al., 2016) and activate COX-2 (Liu et al., 2007), a role for kallikrein-kinin signals that stimulate prostaglandin production may exist in the pathogenesis of SARS-CoV and SARS-CoV-2. The potential for NSAIDs to induce ACE2 production and possibly increase opportunities for SARS-CoV-2 to bind host cell ACE2 has created controversy regarding the use these drugs (Russell et al., 2020). NSAIDs are predominantly non-selective inhibitors of COX-1 activity (Warner and Mitchell, 2004). Classes of NSAIDS include salicylates (e.g. aspirin), salicylic acid derivatives (e.g. 5-aminosalicyclic acid), acetic acid derivatives (e.g. sulindac), oxicams (e.g. piroxicam), propionic acid derivatives (e.g. ibuprofen), and COX-2 inhibitors (e.g. celecoxib). Whether selective COX-2 inhibition has an effect on ACE2 production, SARS-COV-2 replication, or COVID-19 inflammation requires further investigation (**Figure 7**, **Table 3**). Other classes of NSAIDS, aside from aspirin, have beneficial anti-inflammatory effects in general; however, potential cardiovascular effects may limit use in clinical treatment, and are therefore not included in the associated figure and table.

Lastly, bradykinin-induced B2R signals generate IL-6 (Hayashi et al., 2000; Huang et al., 2003) and the activation of B1R or B2R can result in SP production (O’Connor et al., 2004). Both IL-6 and SP participate in the recruitment of neutrophils (Suwa et al., 2001; O’Connor et al., 2004) and the activation of JAK2 cell signals (Hermans et al., 2018; Schett et al., 2020). SP-initiated intracellular signals induce mast cell degranulation (Hermans et al., 2018) and neutrophil respiratory burst activity (O’Connor et al., 2004), suggesting that blocking SP may dampen inflammation. SP also regulates the lung response to inhaled antigens and promotes the pathogenesis of viruses (Munoz and Covenas, 2014). Moreover, cutaneous skin disorders (Vena et al., 2018), altered taste sensitivity (Huang and Wu, 2018), and gastrointestinal disorders (Koon and Pothoulakis, 2006) are also linked to SP as well as COVID-19 (Galvan Casas et al., 2020; Sedaghat et al., 2020; Wong et al., 2020), suggesting that SP may play a role in COVID-19 symptomology. Antagonists to the SP receptor, NK1, are in development for use as antitussive therapy (Smith et al., 2020), which may indicate that SP-activation is involved in the dry cough that is a common in the clinical presentation of COVID-19. The use of inhibitors to bradykinin, SP, IL-6, their receptors, or the downstream signal JAK2 in the kallikrein-kinin system may provide supportive therapy and modulate the COVID-19 inflammatory response in various clinical trials currently in progress (**Table 3**).

## Summary and Implications

SARS-CoV-2 binding to ACE2 is critical to COVID-19 pathophysiological manifestations that develop through the RAAS pathway, the complement system, the coagulation cascade, and the kallikrein-kinin system. The functional disruption of ACE2 by SARS-CoV-2 tends to promote the activation of these pathways, which may already be at a heightened activation state by underlying diseases in patients most susceptible to the virus. The reduced production and activity of ACE2 promotes the formation of Ang II, which in association with SARS-CoV-2-induced DAMPs and PAMPs, promotes the production of complement. This pro-inflammatory response is enhanced by the reduced levels of the ACE2 cleavage product, Ang-(1-7), which is an anti-inflammatory ligand that signals through Mas, MrgD, and AT_2_R (Karnik et al., 2017). Understanding the binding interactions between Ang III and Ang-(1-7) for Mas may be important to the pathophysiology of COVID-19. In addition, both Ang III and SP bind MRGPRX2 (Gembardt et al., 2008; Green et al., 2019). Mast cells are activated by MGRPRX2 ligands and complement fragments (C3a, C5a) (el-Lati et al., 1994; Green et al., 2019). The activity of mast cells is identified in each of these pathways. Specifically, mast cells produce renin and chymase in RAAS, activate complement with renin and tryptase, release heparin and plasminogen factors in the hemostatic system, and produce the pro-inflammatory cytokine IL-6 in response to bradykinin (Theoharides et al., 2011). Mast cells are additionally identified in ARDS and associated with fibroproliferation (Liebler et al., 1998), suggesting that these cells, as well as the more common neutrophils, contribute to ARDS that is a severe and sometimes terminal manifestation in COVID-19 patients (Zhu et al., 2020). Mast cells and their downstream pro-inflammatory mediators may therefore be additional intervention targets in COVID-19.

The activation of COX-2 by SARS-CoV suggests that similar responses may occur with SARS-CoV-2. In addition to cell signals from kallikrein-kinin activation and potential SARS-CoV-2 signals, COX-2 and prostaglandin production can be induced by SP activated NF-kB (Sio et al., 2010) and JAK/STAT (Koon et al., 2006) cell signaling pathways. Prostaglandins promote RAAS activity by stimulating the release of renin, which in turn generates Ang I (Atlas, 2007; Peti-Peterdi and Harris, 2010). SARS-CoV-2 inhibition of ACE2 promotes ACE/Ang II/AT_1_R activity, leading to hypertension-induced SP production (Calvillo et al., 2019). This positive-feedback loop may be supported by the additional release of prostaglandins from neutrophils (Wright et al., 2010) that can also be activated by SP (O’Connor et al., 2004). Thus, because SP is implicated in the pathophysiology of COVID-19 and is a potential factor in nearly all COVID-19 disorders, investigation of interventions that affect SP production and/or functions in COVID-19 patients appears warranted.

Anti-viral therapies will almost certainly be the cornerstone of effective treatments against COVID-19 associated disease. In critically ill patients, additional therapeutic interventions are needed to prevent systemic inflammatory responses that induce organ damage and failure. The physiological manifestations that arise from dysfunction in the RAAS pathway, the complement system, the coagulation cascade, and the kallikrein-kinin system suggest that existing therapeutics along these pathways may be effective in the treatment or mitigation of symptoms in COVID-19 patients.

## Author Contributions

Wrote or contributed to the writing of the manuscript: CC, DR, JK. Illustrations: CC. Developed tables: DR. Edited manuscript: CC, DR, JK.

## Funding

This work was supported by the Intramural Research Program,NIH, Bethesda, MD, including NIDD (ZO1 DK 043308).

## Conflict of Interest

The authors declare that the research was conducted in the absence of any commercial or financial relationships that could be construed as a potential conflict of interest.
